# The Impact of Initial Energy Reserves on Growth Hormone Resistance and Plasma Growth Hormone-Binding Protein Levels in Rainbow Trout Under Feeding and Fasting Conditions

**DOI:** 10.3389/fendo.2018.00231

**Published:** 2018-05-18

**Authors:** Björn Thrandur Björnsson, Ingibjörg Eir Einarsdóttir, Marcus Johansson, Ningping Gong

**Affiliations:** Fish Endocrinology Laboratory, Department of Biological and Environmental Sciences, University of Gothenburg, Gothenburg, Sweden

**Keywords:** adiposity, fasting, salmonid, growth hormone-binding protein, growth hormone resistance

## Abstract

The growth hormone (GH)–insulin-like growth factor I (IGF-I) system regulates important physiological functions in salmonid fish, including hydromineral balance, growth, and metabolism. While major research efforts have been directed toward this complex endocrine system, understanding of some key aspects is lacking. The aim was to provide new insights into GH resistance and growth hormone-binding proteins (GHBPs). Fish frequently respond to catabolic conditions with elevated GH and depressed IGF-I plasma levels, a condition of acquired GH resistance. The underlying mechanisms or the functional significance of GH resistance are, however, not well understood. Although data suggest that a significant proportion of plasma GH is bound to specific GHBPs, the regulation of plasma GHBP levels as well as their role in modulating the GH–IGF-I system in fish is virtually unknown. Two *in vivo* studies were conducted on rainbow trout. In experiment I, fish were fasted for 4 weeks and then refed and sampled over 72 h. In experiment II, two lines of fish with different muscle adiposity were sampled after 1, 2, and 4 weeks of fasting. In both studies, plasma GH, IGF-I, and GHBP levels were assessed as well as the hepatic gene expression of the growth hormone receptor 2a (*ghr2a*) isoform. While most rainbow trout acquired GH resistance within 4 weeks of fasting, fish selected for high muscle adiposity did not. This suggests that GH resistance does not set in while fat reserves as still available for energy metabolism, and that GH resistance is permissive for protein catabolism. Plasma GHBP levels varied between 5 and 25 ng ml^−1^, with large fluctuations during both long-term (4 weeks) fasting and short-term (72 h) refeeding, indicating differentiated responses depending on prior energy status of the fish. The two opposing functions of GHBPs of prolonging the biological half-life of GH while decreasing GH availability to target tissues makes the data interpretation difficult, but nutritional regulatory mechanisms are suggested. The lack of correlation between hepatic *ghr2a* expression and plasma GHBP levels indicate that *ghr2a* assessment cannot be used as a proxy measure for GHBP levels, even if circulating GHBPs are derived from the GH receptor molecule.

## Introduction

As in mammals, the growth hormone (GH)–insulin-like growth factor I (IGF-I) system is the major endocrine system simulating growth in salmonids (1), indicating a strong evolutionary conservation. Indeed, mammalian and salmonid data are in agreement on all key aspects of the GH–IGF-I system. The GH receptor (GHR) is found in most tissues, with the highest density in the liver (2–4). GH can thus stimulate tissue growth directly, but does so also indirectly through GH-induced production of IGF-I in most tissues, where it exerts paracrine regulation of growth and metabolism through its IGF-I receptor (IGF-IR) (1, 5). In the liver, GH stimulates IGF-I secretion into the circulation, where it acts as an endocrine stimulator, especially of skeletal growth (6) as well as acting as a negative-feedback signal on GH secretion (7). In mammals as well as fish, both hormones also regulate important aspects of metabolism through augmentative effects on protein accretion and counteractive effects on glucose and lipid utilization (1, 8, 9).

Fasting normally leads to increased plasma GH levels while IGF-I levels decrease, an endocrine condition defined as acquired GH resistance (10, 11). While early studies in mammals and fish suggested that downregulation of the GHR was the key underlying mechanism (11, 12), it now appears that acquired GH resistance is largely due to inhibition of the JAK–STAT pathway for GH signaling (13–15). Irrespective of the causal mechanisms, one of the major endocrine consequences of GH resistance is the decreased hepatic secretion and thus plasma levels of IGF-I (16). Under catabolic conditions such as fasting, plasma IGF-I levels may decrease independently of the onset of GH resistance, as hepatic IGF-I secretion is stimulated by circulating nutrient levels, which decline during fasting (17). As plasma IGF-I exerts negative-feedback inhibition on pituitary GH secretion (8, 18), decreased IGF-I levels during fasting leads to increased GH secretion and plasma GH levels (19, 20).

Specific binding proteins have been identified and characterized for both GH and IGF-I and therefore, the endocrine regulation of physiological processes by the GH–IGF-I system can be modulated by both growth hormone-binding proteins (GHBPs) and IGFBPs. Much functional information has been obtained on the roles of the multiple IGFBPs, both in mammals and fish (21), while much less is known about the regulatory function of the GHBPs. Despite mammalian data indicating that about 50% of plasma GH is bound to specific, high-affinity GHBP (22–24), its role and impact on GH bioavailability in humans is still unclear (25), as is the functional importance of GHBPs in the GH–IGF-I system in fish.

In mammals, with the exception of rodents (26), the circulating GHBP is the extracellular domain of the GHR protein, which is released into the circulation through proteolytic cleavage of the membrane-bound GHR (27). Thus, the GHR molecule has a double functional role in the GH–IGF-I system; conveying the endocrine GH signal to the target cells as well as being the substrate for plasma GHBP production (28). Mechanistically, observed changes in hepatic GHR density and/or GHR gene expression may indicate changes in tissue sensitivity to GH, and/or changes in GHBP production and plasma GHBP levels.

Sohm et al. (29) provided the first evidence for GHBPs in rainbow trout (*Oncorhynchus mykiss*) plasma using GH binding and cross-linking assays as well as immunoprecipitation and presented semi-quantitative data indicating that plasma GHBP levels increase 2 days after seawater transfer. Similar methodological approach was used to demonstrate the existence of GHBPs in plasma of goldfish (30) and Chinese sturgeon (31). Subsequently, through the use of GHR-transfected CHO cells, Liao et al. (32) demonstrated that fish GHBP stems from the extracellular domain of the membrane-bound GHR, as in non-rodent mammals. Recently, the first immunoassay for non-mammalian vertebrate GHBPs was established and validated for rainbow trout and Atlantic salmon [*Salmo salar* (33)], providing the first ever quantitative data on circulating GHBPs in fish, where plasma GHBP levels were indicated to increase following seawater transfer of Atlantic salmon smolts (33).

The aims of this study were to explore the relation between initial energy balance and the onset of acquired GH resistance by comparing rainbow trout with high and low muscle adiposity during fasting. Furthermore, to gain insights into the regulatory roles of the GH–IGF-I system in rainbow trout in regard to energy balance by elucidating possible roles of plasma GHBPs in functional modulation of the GH–IGF-I system. To achieve these aims, plasma GHBP levels as well as plasma GH and IGF-I levels were measured, together with quantitative analysis of hepatic *ghr2a* mRNA expression, in two separate studies in which the energy balance and nutritional conditions of rainbow trout were manipulated. In experiment I, rainbow trout were fasted for 4 weeks and then refed over 72 h. In experiment II, two selectively bred strains of rainbow trout which differ in muscle and visceral adiposity were studied under feeding conditions as well as during a 4-week fasting period.

## Materials and Methods

### Fish, Holding Conditions, and Design of Experiment I

Rainbow trout (*n* = 116) with a mean body weight (BW) of 146 g and body (fork) length (BL) of 24.7 cm were obtained from a local fish farm, Antens Laxodling AB, outside Gothenburg, Sweden. This stock has been maintained in Swedish aquaculture for generations, but with no directed breeding-selection program. At the animal facilities at the Department of Biological and Environmental Sciences, University of Gothenburg, the fish were randomly distributed among 12 circular 150 l fiberglass tanks, supplied with running, aerated fresh water and acclimated for 2 weeks. Water temperature was 12°C and photoperiod was 12L:12D. The fish were fed manually *ad lib* once a day. After the acclimation period, fish in six tanks were fasted for 4 weeks (FA group) while fish in six tanks were fed *ad lib* during this period (AL group). At the end of the 4-week feeding/fasting period, at time designated as 0 h (*t*_0h_), eight fish from each group were sampled. Then, both AL and FA fish were fed *ad lib* and sampled after 2, 7, 24, and 72 h (*t*_2h_, *t*_7h_, *t*_24h_, and *t*_72h_). Between each sampling, all fish were fed *ad lib* to guarantee maximal feed availability. At each sampling time, eight fish of each treatment regime were sampled, four from two replicate tanks. To minimize disturbance, at least 24 h were allowed to pass before fish were sampled again from a previously sampled tank. The fish were anesthetized with methomidate (12 mg l^−1^), killed by a blow to the head and sampled, see below.

Two of the AL fish sampled at *t*_0h_ had empty gastrointestinal (GI) tract and a low condition factor (CF) similar to that of the FA group. It was concluded that they had not been actively feeding and were eliminated from the study. Data on leptin endocrinology obtained from this study have been presented in Johansson and Björnsson (34).

### Fish, Holding Conditions, and Design of Experiment II

Two divergent rainbow trout lines have been established through a breeding program with muscle adiposity as a selection criterion; a fat line (FL) with high muscle lipid content and a lean line (LL) with low muscle lipid content (35). This study was carried out on fish from the seventh generation of this breeding program These FL fish had double the muscle adiposity of the LL fish, which on the other hand had higher visceral fat content than the FL fish (36). The study was carried out at the PEIMA-INRA aquaculture research facility in Brittany, France. On April 15th, 2014, eight tanks were stocked with FL fish (mean BW 238 g) and eight tanks with LL fish (mean BW 262 g). The water volume of these outdoor tanks was 1.8 m^3^, water flow 3 m^3^ h^−1^ and oxygen levels >6.0 mg l^−1^, under ambient photoperiod and temperature conditions, which rose gradually from 10.6 to 13.5°C over the course of the study from mid-April to early June.

When fed, the fish were given size 5 pellets^1^ by automatic feeders five times daily. The ration was adjusted weekly based on size and temperature, and increased from about 1.16 to 1.25% BW day^−1^ over the study.

After a 3-week acclimation period, a 4-week feeding/fasting experiment was initiated, encompassing four different experimental feeding regimes involving 0, 1, 2, and 4 weeks of fasting. Thus, the 0-week groups were fed throughout, the 1-week groups were fed for 3 weeks followed by 1 week of fasting, the 2-week groups were fed for 2 weeks, and then fasted for 2 weeks, and the 4-week groups were fasted throughout. Each feeding regime included duplicate tanks of FL as well as LL fish. To enable sampling of all fish after 28 days, the experimental feeding regimes were initiated 1 day apart and then sampled 1 day apart 4 weeks later.

For each feeding regime, 20 fish of each line were sampled, 10 from each of the duplicate tanks. The fish were netted and placed in a lethal dose (160 mg l^−1^) of isoeugenol (ScanAqua). When ventilation ceased, the fish were sampled, see below.

Data from this experiment on peripheral leptin endocrinology and energy stores have been published in Johansson et al. (36) and on central leptin signaling in Gong et al. (37).

### Sampling

Sampling was initiated by measurements of BW and BL, after which blood was drawn from the caudal vessels into a heparinized syringe. Blood was kept on ice for <15 min before centrifuged, the obtained plasma frozen in aliquots on dry ice and kept at −80°C until analysis. The liver was dissected out and weighted (LW), after which about 1 g piece was placed in aluminum foil, immediately frozen in liquid nitrogen and kept at −80°C until analysis. The whole GI tract was dissected out and weighted after which all food was removed from both stomach and intestine and it weighted again as visceral weight (VW).

### Analyses

#### Plasma GH and IGF-I Analysis

Plasma GH was analyzed with radioimmunoassay (RIA), using anti-GH antibodies specific for salmonids. The method has been described by Björnsson et al. (38) and evaluated for rainbow trout.

IGF-I was extracted from plasma as described by Shimizu et al. (39) and analyzed using a 2-day RIA protocol described by GroPep Ltd.^2^ with some modifications. Salmon/trout IGF-I (GroPep) was used for iodination and standards. Antibodies against barramundi IGF-I, obtained from GroPep, were made in rabbits by Agrisera.^3^ Iodination was carried out using chloramine-T, with 0.5% BSA added to the RIA buffer. Microliters of the extracted neutralized samples were diluted 1:4 with RIA buffer, and 100 µl samples and standards were analyzed. Anti-barramundi IGF-I rabbit serum was used at a final dilution of 1:42,000 in the assay tubes, and the assay ^125^I-IGF-I solution was adjusted to 5,000 cpm per 50 µl solution. The antigen–antibody complex was precipitated with anti-rabbit IgG (R0881), and gamma globulin (I 8140) from Sigma^4^ and 3% polyethylene glycol (PEG 6000) After incubation, the samples were centrifuged at 3,200 rpm for 60 min, aspirated and the pellets counted in a gamma counter.

#### Plasma GHBP Analysis

Plasma GHBP was analyzed using a 3-day competitive, non-equilibrium RIA, described and validated for rainbow trout by Einarsdottir et al. (33). Briefly, a GST-tagged recombinant extracellular part of the Atlantic salmon GHR subtype 1 (sGHR1) was used as standards and iodinated with the chloramine-T method. Antibody against the extracellular part of the sGHR1 (anti-sGHR1) was produced in rabbits by Agrisera against a 15 amino acid synthetic peptide conjugated to keyhole limpet hemocyanin. The rabbit anti-GHR1 serum was affinity purified with the antigen coupled to the stationary phase. The sGHR1 isoform, described by Benedet et al. (40), corresponds to the rainbow trout GHR2a isoform as it is defined by Reindl and Sheridan (3) and was previously termed GHR1, and the synthesized amino acid sequence used to raise the anti-sGHR1 is near-identical between the two species.

#### Hepatic Growth Hormone Receptor 2a (*ghr2a*) Gene Expression Analysis

Total RNA was extracted from 30 mg liver using RNeasy^®^ Plus Mini Kit (Qiagen, Hilden, Germany). For the tissue homogenizing, TissueLyser II (Qiagen) was used in Study I, with each tube containing a 5 mm ∅ stainless steel bead (Qiagen), and in Study II, the Precellys^®^24 homogenizer (Bertin Technologies, France) was used. RNA quantity and quality were assessed using the NanoDrop Spectrophotometer (Thermo Fisher Scientific, Wilmington, DE, USA) by absorbance at 260 and 280 nm. To check the RNA quality with another method, random samples were assessed from Study II using the Experion Automated Electrophoresis System (Bio-Rad Laboratories, Sundbyberg, Sweden). Total RNA (1 µg) was reverse-transcribed to cDNA using iScript™ cDNA Synthesis Kit on a MyCycler Thermal Cycler (Bio-Rad). The GHR isoform analyzed has earlier been termed GHR1 [e.g., Ref. (9, 14)], but has since been redefined as GHR2a (3). The gene expression of the rainbow trout *ghr2a* and reference gene, elongation factor 1α (*elfl1*α) was analyzed by quantitative PCR (qPCR) using the reagent, SsoAdvanced™ Universal SYBR^®^ Green supermix (Bio-Rad), in a CFX Connect™ real-time cycler (Bio-Rad). The primer sequences were listed in Table 1, and purchased from Eurofins MWG Synthesis GmbH, Ebersberg, Germany.

**Table 1:** Primer nucleotide sequences used for quantitative PCR analysis of rainbow trout hepatic growth hormone receptor gene 2a (*ghr2a*) and reference gene *elf1*α in experiments I and II.

Gene	Primer	Sequence (5′–3′)
*ghr2a*^a^	GHR2aFwOm	TGGGAAGATGAGTGCCAGACT
	GHR2aReOm	CACAAGACTACTGTCCTCTGTTGG

*elf1*α	EFa-f	CAAGGATATCCGTCGTGGCA
	EFa-r	ACAGCGAAACGACCAAGAGG

*^a^The *ghr2a* gene was earlier termed *ghr1*, see Ref. (3)*.

All samples were analyzed in duplicate using 5 µl qPCR reagent, 0.5 µl of each primer (final concentration 500 nM), and 4 µl cDNA template (10 ng cDNA) in a total volume of 10 µl. The qPCR reaction involved 40 cycles using a dissociation temperature of 95°C for 10 s and annealing and elongation in the same step at 60°C for 30 s. The quantification cycle number (*C*_q_) was used to calculate the gene expression for each sample. No non-specific products or primer-dimers were co-amplified with the specific product. Both target gene (*ghr2a*) and reference gene (*elf1*α) was amplified with efficiencies near 100%.

### Ethical Permits

Experiment I was carried out at a certified animal facility at the Department of Biological and Environmental Sciences, University of Gothenburg, under license 85-2012 by the Ethical Committee for Animal Research in Gothenburg. Experiment II was carried out at Pisciculture Expérimentale INRA des Monts d’Arrée, which is approved for animal experimentation through license C29-277-02 in accordance with the European Communities Council Directive 86/609/EEC, and carried out under the official license 29-036 of Dr. Labbé Laurent.

### Calculations and Statistics

Condition factor was calculated as CF = (BW × BL^−3^) × 100. Liver somatic index (LSI) was calculated as LSI = (LW × BW^−1^) × 100, and visceral somatic index (VSI) was calculated as VSI = (VW × BW^−1^) × 100. For the calculations, all weights (BW, LW, and VW) are expressed in grams, and BL is expressed in centimeters.

The relative hepatic expression level of the *ghr2a* gene was calculated using the formula of ratio (target/reference) = 2^Cq (reference) − Cq (target)^.

The data from experiments I and II were statistically analyzed using two-way ANOVA, establishing the significance of the main effects (feeding regime and time in experiment I; line and time in experiment II) as well as the interaction between the main effects. When main effects were found to be statistically significant, *post hoc* analysis was conducted using Fisher’s least significant differences. The statistical analysis was carried out using the IBM SPSS Statistics version 25 software package.

## Results

### Experiment I

**Figure 1:**
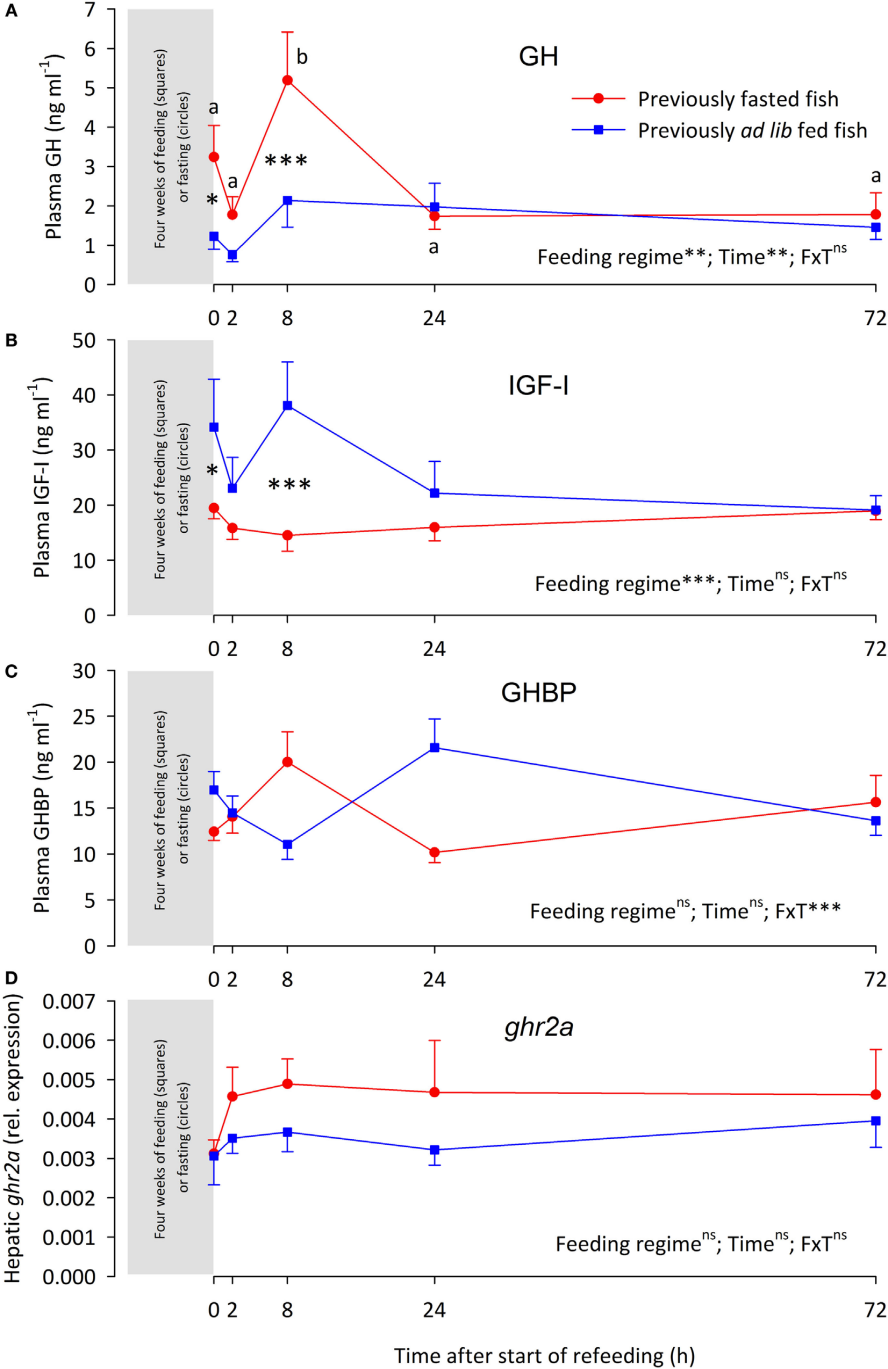
**(A)** Plasma growth hormone (GH) levels, **(B)** plasma insulin-like growth factor I (IGF-I) levels, **(C)** plasma growth hormone-binding protein (GHBP) levels, and **(D)** hepatic expression of the growth hormone receptor gene 2a (*ghr2a*) in rainbow trout fed (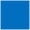) or fasted (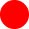) for 4 weeks (sampled at time 0 h) after which both groups were fed over a 72 h “refeeding” period. Data are presented as means ± SEM. Two-way ANOVA results on main effects (feeding regime and time) as well as the interaction between the main effects (F × T) are indicated in the panels as being non-significant (ns, *p* > 0.05) or significant at the levels of **p < 0.01 and ***p < 0.001. *Post hoc* analysis was carried out if main effects were significant. For “Feeding regime,” differences are indicated as *p < 0.05 or ***p < 0.001. For “time,” significant differences are indicated by different letters, lower case for fasted fish.

At the end of the 4-week fasting period (*t*_0h_), the FA fish had significantly lower BW, CF, LSI, and VSI than the AL fish (Table 2). Furthermore, in comparison with the AL fish, the FA fish had elevated plasma GH levels (Figure 1A), suppressed plasma IGF-I (Figure 1B), while plasma GHBP levels (Figure 1C) and hepatic *ghr2a* expression was similar between the groups (Figure 1D).

**Table 2:** Body weight (BW), condition factor (CF), liver somatic index (LSI), and visceral somatic index (VSI) of rainbow trout fed *ad lib* (AL group; *n* = 6) or fasted (FA group; *n* = 7) for 4 weeks in experiment I and sampled before onset of refeeding.

Group	BW (g)	CF	LSI (%)	VSI (%)
Fed *ad lib (*AL)	227.4 ± 26.6	1.33 ± 0.08	1.26 ± 0.14	1.55 ± 0.11
Fasted (FA)	146.3 ± 5.2*	1.06 ± 0.04*	0.62 ± 0.06*	1.04 ± 0.07*

*Data are presented as means ± SEM*.

*Statistical significant differences between groups at the p < 0.05 level.

Refeeding differentially affected the various components of the GH–IGF-I system. Thus, plasma GH levels of the FA fish were elevated over the AL fish at 8 h, after which plasma GH levels were similar in both groups (Figure 1A). Conversely, plasma IGF-I levels of the FA fish were depressed at 8 h, after plasma IGF-I levels were similar between the groups (Figure 1B). Plasma GHBP levels oscillated. While neither feeding regime nor time significantly affected GHBP levels, the interaction of the main effects was significant (Figure 1C). The hepatic *ghr2a* expression remained similar between the groups over the 72 h refeeding period (Figure 1D).

Correlation analysis of plasma GH, IGF-I, and GHBP levels as well as hepatic *ghr2a* expression shows no significant correlation among these parameters (data not shown).

### Experiment II

**Figure 2:**
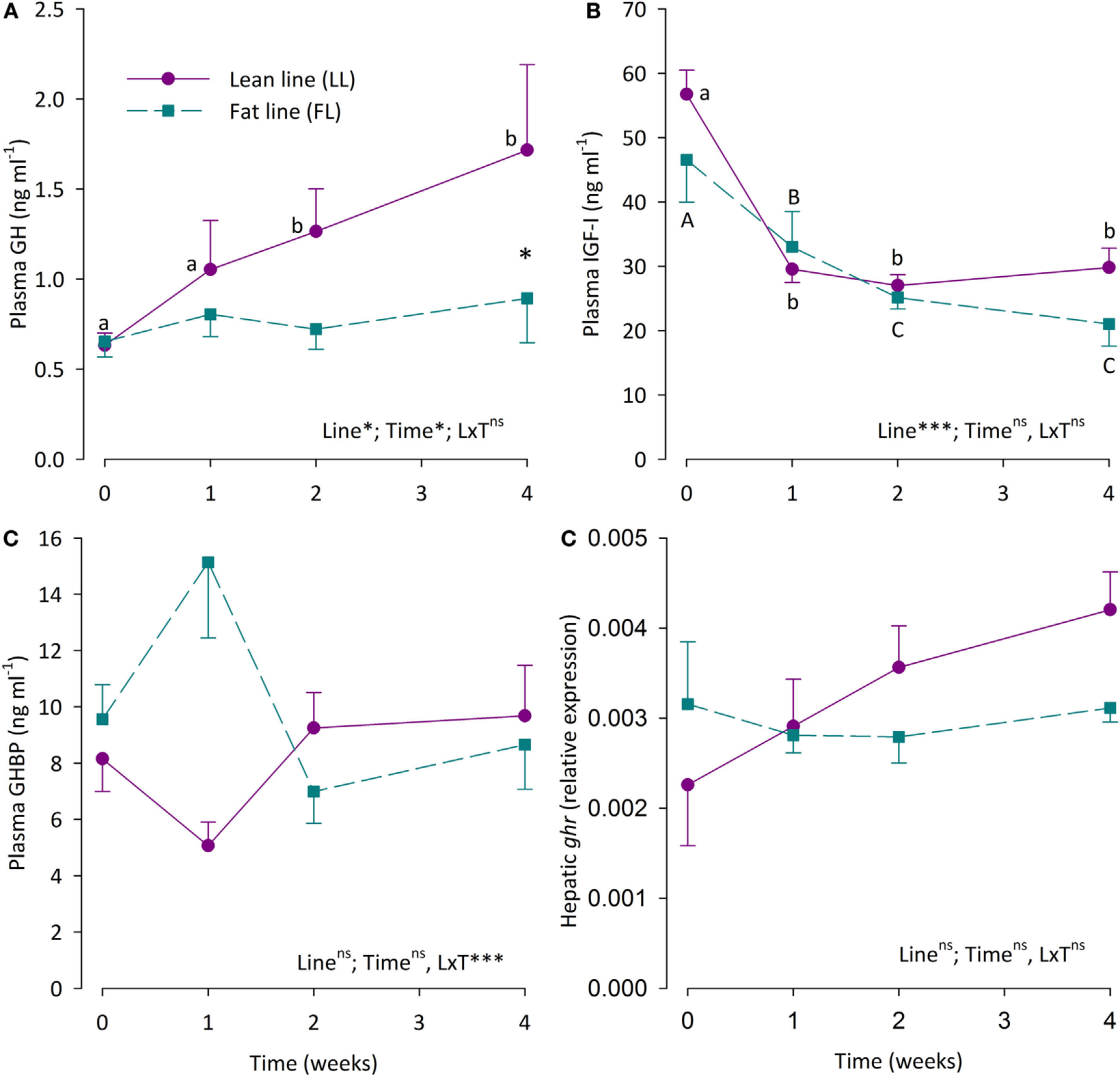
**(A)** Plasma growth hormone (GH) levels, **(B)** plasma insulin-like growth factor I (IGF-I) levels, **(C)** plasma growth hormone-binding protein (GHBP) levels, and **(D)** hepatic expression of the growth hormone receptor gene 2a (*ghr2a*) in fat line (FL, 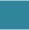) and lean line (LL, 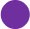) rainbow trout over a 4-week fasting period. Data are presented as means ± SEM. Two-way ANOVA results on main effects (strain and time) as well as the interaction between the main effects (S × T) are indicated in the panels as being non-significant (ns, *p* > 0.05) or significant at the levels of *p < 0.05, or ***p < 0.001. *Post hoc* analysis was carried out if main effects were significant. For “Strain,” differences are indicated as *p < 0.05. For “Time,” significant differences are indicated by different letters, lower case for LL fish and upper case for FL fish.

Plasma levels of GH, IGF-I, and GHBP, as well as hepatic *ghr2a* expression in the LL and the FL fish are shown in Figure 2. Plasma GH levels were significantly elevated in LL fish during fasting, being significantly elevated after 2 and 4 weeks, while plasma GH levels of the FL fish were not affected by fasting. Thus, after 4 weeks of fasting, the LL fish had significantly higher plasma GH levels than the FL fish. Plasma IGF-I levels decreased successively in a similar manner in both fish groups during fasting and were significantly lower than pre-fasting levels already after 1 week of fasting. Plasma GHBP levels oscillated. While neither fish line nor time significantly affected GHBP levels, the interaction of the main effects was significant. Relative hepatic *ghr2a* gene expression did not differ statistically between the fish lines or different fasting periods.

Correlation analysis of plasma GH, IGF-I, and GHBP levels as well as hepatic *ghr2a* expression shows no significant correlation among these parameters (data not shown).

Energy reserves as reflected in LSI, VSI, and muscle fat content are given in Table 3. LSI was similar between FL and LL fish and decreased significantly during the first week of fasting after which it did not decrease further in either group. VSI was higher in LL than FL fish throughout the 4-week fasting, during which the VSI declined successively in both groups. Muscle lipid levels were higher in FL than LL fish both initially and after 1-week fasting. However, while muscle lipid levels did not change in the LL fish throughout the fasting period, they declined continuously in the FL group.

**Table 3:** Energy-related physical characteristics of fat line (FL) and lean line (LL) rainbow trout of experiment II under normal feeding conditions (0-week fasting), and after 1, 2, and 4 weeks of fasting.

Fasting (weeks)	LSI (%)		VSI (%)		Muscle lipid content (%)	
	FL	LL	FL	LL	FL	LL
0	1.23 ± 0.05^A^	1.16 ± 0.03^A^	8.24 ± 0.31^A,^**	9.67 ± 0.23^A^	7.31 ± 0.72^A,^**	3.90 ± 0.54^A^
1	0.85 ± 0.02^B^	0.88 ± 0.04^B^	7.02 ± 0.39^B,^**	8.22 ± 0.24^B^	5.10 ± 0.84^A,B,^**	3.06 ± 0.39^A^
2	0.79 ± 0.05^B^	0.81 ± 0.03^B^	6.69 ± 0.16^B,C,^**	7.95 ± 0.22^B,C^	4.58 ± 0.58^B,C^	2.99 ± 0.40^A^
4	0.75 ± 0.03^B,^**	0.89 ± 0.03^B^	5.71 ± 0.26^C,^**	7.09 ± 0.37^C^	3.26 ± 0.22^C^	3.43 ± 0.62^A^

*These include liver somatic index (LSI), visceral somatic index (VSI), and muscle lipid content*.

Statistical significant differences between groups are indicated as *p < 0.05 or **p < 0.01.

*Statistical significant differences (*p* < 0.05) over time are indicated with different superscript letters*.

## Discussion

This study allows examination of two important aspects of the GH–IGF-I system in rainbow trout, i.e., the functional importance of GH resistance in energy mobilization during fasting, and whether plasma GHBPs are exerting a modulating effect on the GH–IGF-I system during fasting and refeeding. Furthermore, the study explores the short-term responses of the GH–IGF-I system during refeeding.

### GH Resistance and Energy Mobilization in Rainbow Trout During Fasting

In this study, the rainbow trout which have been breeding-selected for high muscle adiposity for seven generations (FL fish of experiment II) do not, in contrast to the other rainbow trout studied, enter a state of acquired GH resistance during the 4-week fasting, as they maintain normal GH levels. This is most likely linked to the high, initial energy reserves of these fish, allowing them to mobilize lipids throughout the 4-week fasting period. As described in Johansson et al. (36), while visceral lipids were mobilized to similar extent in both fish lines [VSI %, 0 → 4 weeks of fasting; FL: 8.24 → 5.71 (−30.7%), LL: 9.67 → 7.09 (−26.7%)], the FL fish were able to continuously mobilize muscle fat over the 4-week fasting period. By contrast, the LL fish did not have enough initial muscle fat reserves to mobilize significant amounts of fat [muscle lipid content %, FL 7.31 → 3.26 (−52.4%), LL: 3.90 → 3.43 (−12.1%)]. Thus, during the fasting period, the LL fish have probably activated protein-dominated catabolism, which occurs in vertebrates only when the lipid levels have reached a critical threshold, with proteins being the fuel of last resort during starvation (41). As GH resistance is the key endocrine mechanism permitting protein catabolism (42), the GH resistance in the LL fish may act as a permissive mechanism, allowing the fish to meet metabolic demands during fasting through protein catabolism when carbohydrate and lipid reserves are depleted.

Although the GH part of the GH–IGF-I system is functioning normally in the FL fish, with normal hepatic *ghr2a* expression and normal plasma GH levels, the IGF-I part of the system is suppressed during fasting. This is likely to be linked to some aspects of the catabolic state the fish are in, as IGF-I expression and plasma levels in both fish and mammals are strongly affected by nutritional status, with fasting leading to depressed IGF-I activity (43–46).

The elevated plasma GH levels during fasting in the fish of experiment I (FA group) and the LL fish of experiment II are a response frequently observed during fasting in salmonids (47–50). Together with declining plasma IGF-I levels, this indicates that the fish are entering a state of acquired GH resistance, a state typically observed under catabolic conditions such as fasting (11, 42) in various vertebrate groups (44), including fish (17).

It has been hypothesized that the primary mechanism for this condition to develop is a downregulation of the hepatic GHR, leading to decreased plasma IGF-I levels and thus decreased IGF-I feedback inhibition of pituitary GH secretion, resulting in elevated plasma GH levels (12, 51). Thus, this hypothesis postulates that fasting-induced elevation of plasma GH levels is be due to an increased pituitary GH secretion rate as well as decreased hepatic clearance rate of the hormone. However, the GH resistant rainbow trout in this study shows no indication of hepatic GHR downregulation after 4-week fasting as *ghr2a* mRNA levels remain unchanged. Although care should be taken not to overinterpret gene expression data as they are not a very reliable indicator of protein abundance (52), the current data do not support this hypothesis. The present data are in agreement with a study by Norbeck et al. (9) in which hepatic *ghr2a* (then termed GHR1) expression was unaffected by 2-week fasting. However, that study also included rainbow trout fasted for 6 weeks, at which time-point the hepatic *ghr2a* expression was suppressed. By contrast, rainbow trout fasted for 30 days in a study by Gabillard et al. (14) had significantly elevated hepatic *ghr2a* mRNA abundance. Thus, data on the GH–IGF-I system in rainbow trout during fasting diverge in terms of the effects on hepatic *ghr* expression. In mammals, starvation can decrease GHR levels while malnutrition such as protein deficiency rather appears to inhibit the post-GHR signaling pathways (10, 13, 46). This indicates that the severity of the fasting/starvation episode will affect the outcome in terms of hepatic GHR expression and density. As both water temperatures and initial energy reserves will influence the temporal severity of fasting in fish, these factors may lead to the divergent hepatic *ghr* expression observed in the rainbow trout [this study; (9, 14)].

While speculative, as GH secretion and clearance rates have not been assessed in this study, it appears likely that in the absence of hepatic GHR downregulation, elevated GH levels in fasting fish are primarily due to increased pituitary GH secretion rate.

### Regulation of Plasma GHBP Levels During Fasting and Refeeding

In fish as in most mammals, GHBPs are principally generated through proteolysis of the full-length GHR (32, 53, 54), rather than through alternative splicing of the *ghr* gene, as in rodents (55, 56). However, truncated *ghr* genes encoding for the extracellular GHR domain have been identified in both early and late vertebrates such as sea lamprey (57) and human (53) as well as zebrafish (58), representing an alternative production pathway for plasma GHBPs.

The high GHR density in the salmonid liver (2, 25) makes it a likely organ source for plasma GHBP levels. It could thus be suggested that correlation existed between *ghr2a* levels and plasma GHBP levels in the rainbow trout. This study clearly demonstrates that this is not the case, as also has been observed in humans (25), indicating that hepatic *ghr* expression is not a reliable predictor of plasma GHBP levels. Such lack of correlation is not surprising, as the circulating GHBP levels are dependent on the posttranslational cleavage of the extracellular domain of the GHR, an enzymatic mechanism which is independently regulated, making the *ghr*–GHBP link even less direct (59).

In both experiments, plasma GHBP levels fluctuated with time in such a way that while no main effects of feeding regime (experiment I) or fasting (experiment II) were found, there was significant interaction between the main effects. This indicates differentiated regulation of plasma GHBP levels, both during fasting and refeeding, based on the prior energetic status of the fish established through feeding regime in experiment I and breeding selection in experiment II.

Thus, the current GHBP data are complex and make it hard to propose a defined regulatory role for circulating GHBPs in the endocrine GH–IGF-I system in rainbow trout. This echoes conclusions from mammalian studies. By binding GH, plasma GHBPs prolong the biological half-life of the hormone, but at the same time decrease availability of GH to target tissues through competing GHR ligation and limit the free GH levels. These two opposing mechanisms through which GHBPs affect GH kinetics has made it hard to establish the role of GHBPs and their impact on GH bioavailability in mammals, including humans (25).

However, it appears likely that plasma GHBP levels in the rainbow trout are to some extent regulated by nutritional factors, as seen in the rat (60). Furthermore, the relatively elevated GHBP levels in FL fish after 1-week fasting, concomitant with low plasma GH levels, suggest that the GH-endocrinology has been altered during the genetic selection for high muscle adiposity, and the FL fish may represent an “obesity” phenotype (37), similar as seen in obese humans with low GH and high GHBP plasma levels (61, 62).

### Short-Term Impact of Refeeding on the GH–IGF-I System

The initiation of refeeding after 4-week fasting of rainbow trout in experiment I leads to relatively rapid (2–24 h) changes in plasma levels of GH, IGF-I and GHBP, i.e., the components of the GH–IGF-I system which had previously been affected by the fasting. This suggests that the GH–IGF-I system is rapidly readjusting, and that a shift in the endocrine regulation of growth and energy balance from catabolic to anabolic conditions is completed within 72 h of the onset of refeeding. The present hormonal data are in line with earlier data on “corrective” shifts in plasma GH and IGF-I levels during refeeding of rainbow trout (9, 14) and fine flounder (20), even if these studies indicate that while plasma GH levels reach “normal” pre-fasting levels within days, it may take as long as 2 weeks for plasma IGF-I levels to normalize.

As the hepatic *ghr2a* expression was unaffected by the 4-week fasting and was similarly unaffected by refeeding, the gene expression of this GHR isoform does not appear to be a major regulatory component of the GH–IGF-I system, even if downregulation (63) as well as upregulation (14) of this gene has been reported during fasting of salmonids.

### Conclusion and Future Perspectives

The causal mechanisms and functional significance of acquired GH resistance during fasting in fish has received limited attention. This study provides a novel experimental model. It shows that manipulation of energy reserves, such as through breeding selection for high muscle adiposity, can affect whether or not acquired GH resistance sets in during a period of fasting. As the physiological function of GH in salmonids as in other vertebrates is to favor protein synthesis over break-down, the functional significance of GH resistance during fasting is likely to allow protein catabolism to proceed when lipid stores are depleted. In this context, the link between GH resistance and protein catabolism needs to be studied further. It can, e.g., be hypothesized that if the FL fish had been fasted for longer than 4 weeks, at which point they had little or no muscle fat reserves left to mobilize, the fish would develop GH resistance and enter a starvation phase of muscle protein break-down.

Although this study provides novel data on plasma GHBP levels in salmonids and non-mammalian vertebrates in general, current understanding on mechanisms regulating GHBP levels as well as the functional significance of plasma GHBPs as modulators of the GH–IGF-I system is still severely lacking. This study demonstrates that analysis of hepatic *ghr2a* expression does not provide a useful proxy measure for plasma GHBP levels, as correlation between these parameters is lacking. Thus, direct measurements of circulating GHBPs appear necessary, and thus, the RIA established by Einarsdottir et al. (33) is a major step forward.

The current data indicate that GHBP may be nutritionally regulated and could possibly act as a temporary modulator of GH action during postprandial periods and short-term fasting, but further studies are clearly needed in this area. Future studies on fish should, e.g., explore the activity and regulation of the proteases responsible for GHBP production to elucidate if they represent an important regulatory mechanism.

## Ethics Statement

Experiment I was carried out at a certified animal facility at the Department of Biological and Environmental Sciences, University of Gothenburg, under license 85-2012 by the Ethical Committee for Animal Research in Gothenburg. Experiment II was carried out at Pisciculture Expérimentale INRA des Monts d’Arrée, which is approved for animal experimentation through license C29-277-02 in accordance with the European Communities Council Directive 86/609/EEC, and carried out under the official license 29-036 of Dr. Labbé Laurent.

## Author Contributions

MJ, NG, IE, and BB designed and carried out the experiments. IE and MJ carried out the sample assays and data analysis. IE, BB, and NG are responsible for the writing.

## Conflict of Interest Statement

The authors declare that the research was conducted in the absence of any commercial or financial relationships that could be construed as a potential conflict of interest.

## Footnotes

^1^www.aqua.legouessant.com.

^2^http://gropep.com.

^3^http://agrisera.com.

^4^http://sigmaaldrich.com.
